# The AViKA (Adding Value in Knee Arthroplasty) postoperative care navigation trial: rationale and design features

**DOI:** 10.1186/1471-2474-14-290

**Published:** 2013-10-12

**Authors:** Elena Losina, Jamie E Collins, Meghan E Daigle, Laurel A Donnell-Fink, Julian JZ Prokopetz, Doris Strnad, Vladislav Lerner, Benjamin N Rome, Roya Ghazinouri, Debra J Skoniecki, Jeffrey N Katz, John Wright

**Affiliations:** 1 Department of Orthopedic Surgery, Brigham and Women’s Hospital, Boston, MA 02115, USA; 2 Harvard Medical School, Harvard University, Boston, MA 02115, USA; 3 Orthopaedic and Arthritis Center for Outcomes Research, Brigham and Women’s Hospital, Boston, MA 02115, USA; 4 Department of Biostatistics, Boston University School of Public Health, Boston, MA 02118, USA

**Keywords:** Total knee arthroplasty, Motivational interviewing, Functional status, Pain

## Abstract

**Background:**

Utilization of total knee arthroplasty is increasing rapidly. A substantial number of total knee arthroplasty recipients have persistent pain after surgery. Our objective was to design a randomized controlled trial to establish the efficacy of a motivational-interviewing-based telephone intervention aimed at improving patient outcomes and satisfaction following total knee arthroplasty.

**Methods/Design:**

The study was conducted at Brigham and Women’s Hospital in Boston, Massachusetts. The study focused on individuals 40 years or older with a primary diagnosis of osteoarthritis who were scheduled for total knee arthroplasty. The study compared two management strategies over the first six months postoperatively: 1) enhanced postoperative care with frequent follow-up by a care navigator; 2) usual postoperative care. Those who were randomized into the enhanced postoperative care arm received ten calls from a trained non-clinician care navigator over the first six postoperative months. The navigator used motivational interviewing techniques to engage patients in discussions related to their rehabilitation goals, including patient’s plans for and confidence in achieving those goals. Patients in the usual care arm received standard postoperative management and received no navigator phone calls. Patients in both arms were assessed at baseline, three months, and six months postoperatively.

**Discussion:**

The primary outcome of the study was improvement in function as measured by the difference in Western Ontario and McMaster Universities Osteoarthritis Index function score between preoperative (baseline) status and six months postoperatively. Data were collected to identify factors that may be related to total knee arthroplasty outcomes, including preoperative pain, pain catastrophizing, self-efficacy, and depression. A formal economic analysis is also planned to determine the cost-effectiveness of the care navigator as a component of total knee arthroplasty care.

**Trial registration:**

ClinicalTrials.gov NCT01540851

## Background

Total knee arthroplasty (TKA) is a frequently used procedure to reduce pain, improve functional status, and enhance quality of life in persons affected by advanced knee osteoarthritis (OA) [1]. A growing body of evidence suggests that approximately 20% of TKA recipients have persistent knee pain following surgery [2]. Greater preoperative levels of pain, functional limitation, medical comorbidity, depression, catastrophizing and lower self-efficacy have all been identified as potential risk factors for poor symptomatic and functional outcomes of TKA [3-7].

Motivational interviewing (MI) is a client-centered, semi-directive method of engaging intrinsic motivation to change behavior. MI was developed in the context of substance abuse, but has since been applied to a variety of health problems ranging from unsafe sexual behavior to diabetes management [8,9]. Rosal and colleagues designed a randomized trial to establish the efficacy of a theory-based telephone-delivered short Patient Self-Management Support intervention in post-TKA patients [10]. The intervention was partially based on the principles of MI and concluded 9 weeks post-TKA. Functional outcomes were measured at 6 and 12 months post-TKA. The preliminary results presented at the Annual Meeting of the American Academy of Orthopedic Surgeons in 2012 did not show a substantial effect of the intervention on functional improvement. However, the rehabilitation process following TKA is rigorous and often takes longer than 8–9 weeks [11]. We aim to build upon the Rosal trial by using an MI-based intervention, extending the intervention to 22 weeks and gathering additional data on adherence to the rehabilitative process, utilization of emergency departments, and satisfaction with the surgery.

The Adding Value in Knee Arthroplasty (AViKA) Postoperative Care Navigation Trial is a randomized controlled trial to assess the efficacy of an MI-based postoperative intervention, carried out by MI-trained researchers (“navigators”), on the functional recovery of patients undergoing primary unilateral TKA for OA.

## Methods/Design

The team considered retrospective vs. prospective data collection and observational vs. interventional designs. Retrospective studies are typically less costly and time-consuming than prospective studies. However, a retrospective design could not address our research question because the current standard of care practices for TKA patients does not include the motivational interviewing (MI) intervention we sought to evaluate. We considered using a single-arm study of a navigator-led intervention and comparing the results with historical controls or control patients at another institution. However, the processes of care for TKA recipients are evolving rapidly and are not uniform across centers. Thus, the contrast between intervention and control could be complicated by secular and institutional differences in the care process. Equally important, non-randomized designs do not ensure balance in patient characteristics across treatment arms.

The randomized controlled trial (RCT) overcomes these problems, but may introduce its own limitations. First, an RCT may lack external validity if a substantial number of patients refuse to participate, raising the question of whether the participants truly represent the population that the findings are intended to inform. However we discussed the RCT design with post-TKA patients in focus groups who indicated they would be comfortable with randomization in this setting. On the basis of all these considerations, we proceeded with a two-arm randomized controlled trial. To address the issue of external validity and generalizability, we collected basic demographic baseline data on patients who were eligible but chose not to participate.

Patients randomized to the navigator arm received an intervention based on MI principles with a focus on behavior change. Behavior change has been described using the Transtheoretical Model, which distinguishes six phases of change: pre-contemplation, contemplation, determination, action, maintenance, and relapse [12,13]. The Adding Value in Knee Arthroplasty (AViKA) care navigator intervention is focused on behavior change in three domains: adherence to prescribed physical therapy, increased weekly physical activity, and achievement of patients’ personal goals for recovery.

### Setting and sample

#### Ethics statement

This study was approved by the Partners Healthcare Institutional Review Board (IRB) (protocol 2010P002597). Written consent was obtained from all participants in the AViKA Trial.

The AViKA Trial was conducted at Brigham and Women’s Hospital (BWH), a tertiary academic medical center in Boston, MA. The surgeons in the BWH Department of Orthopedic Surgery perform over 1,400 TKAs annually. Patients were recruited from the practices of five BWH orthopedic surgeons. To be eligible to participate in AViKA, patients needed to be 40 years of age or older, scheduled for a primary TKA at BWH, and have a diagnosis of degenerative knee OA. The full inclusion and exclusion criteria (including justifications for these criteria) are provided in Table 1.

**Table 1:** AViKA study inclusion and exclusion criteria

**Inclusion Criteria:**	**Exclusion Criteria:**
▪ Scheduled to undergo primary TKA at BWH	▪ Osteoarthritis is not the underlying diagnosis (e.g. inflammatory arthritis)
▪ Osteoarthritis is the principal underlying diagnosis	▪ Psychological issues that preclude participation, as identified by participating surgeons
▪ Age ≥ 40 years at the projected date of TKA	▪ Dementia
▪ English-speaking	
	▪ Non-English speaker
	▪ Age < 40 at the projected date of TKA
	▪ Lives in a nursing home (difficult to track costs)
	▪ Implantation of unicompartmental knee arthroplasty or interpositional arthroplasty (different clinical features and different costs)
	▪ Bilateral TKA (simultaneous, staged or planned within 6 months)

### Screening, enrollment, and randomization

#### Screening

Patient eligibility was assessed by a research assistant using two data sources, the Partners HealthCare Longitudinal Medical Record (LMR) and Electronic Surgical Plan (ESP). Upon confirming basic patient eligibility in LMR and ESP, operating surgeons were sent a confidential email listing potentially eligible patients and their date of surgery. Surgeons were asked to exclude patients with dementia or psychological issues that would preclude participation, patients with planned unicompartmental knee arthroplasty, interpositional arthroplasty, or bilateral (staged or simultaneous) TKA. Patients were considered to have passed the surgeon screen if the operating surgeon did not request exclusion from the study within three business days.

#### Enrollment

The Partners IRB required that a recruitment letter be sent to each patient before enrollment in the trial. The recruitment letter described the goals of the study, explained randomization and the intervention and detailed the payment plan for participants. Patients were informed that they would be called by the research assistant and invited to enroll in the study if they did not actively opt out prior to the enrollment call. Due to time constraints, patients with TKA scheduled within 28 days of the screening date were not eligible for recruitment unless the operating surgeon provided written confirmation that the study had been discussed during the clinic visit.

Patients who did not opt out of the study were contacted by a research assistant, who used a standardized script to provide a detailed description of the study. Patients who wished to participate were mailed an informed consent form and baseline questionnaire. The research assistant then met patients in person during their standard preoperative appointment (usually scheduled two weeks before surgery) to obtain written informed consent and collect the baseline questionnaire. For the first 9 months of the study the research assistant also performed a brief physical examination during the baseline visit that measured knee range of motion.

#### Randomization

**Figure 1:**
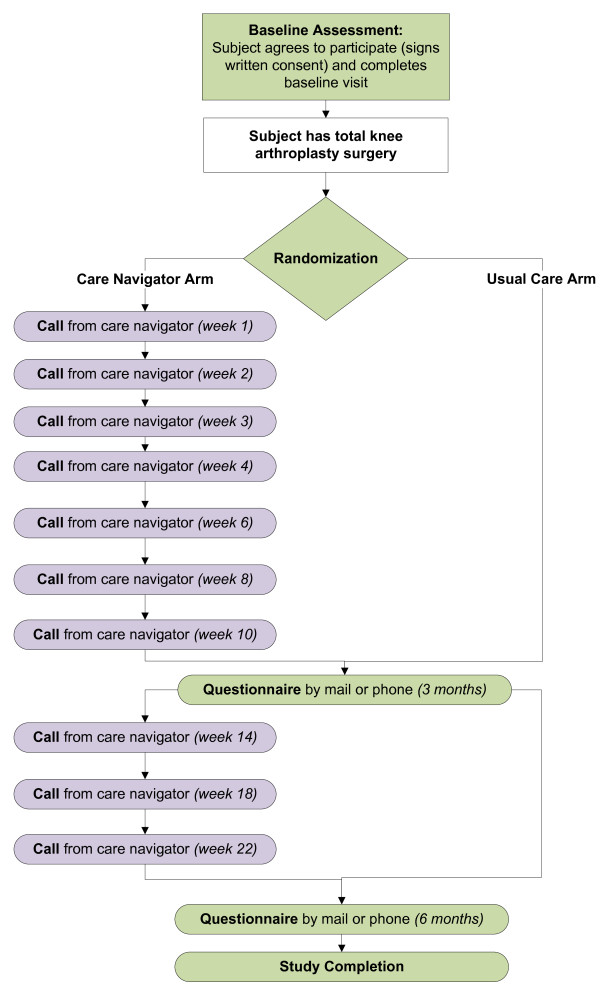
**AViKA treatment arms.** This figure depicts the two treatment arms of the AViKA study: the care navigator arm and the usual care arm. Patients in both arms received questionnaires at baseline and post-operative months three and six. Patients in the care navigator arm also receive regular phone calls from the care navigator. The time points designated in parenthesis indicate the post-operative time point (that is, “Call from care navigator (*week 4*)” indicates that the care navigator called the patient one month after the date of the patient’s TKA).

After surgery, patients were randomized to one of the two treatment arms, enhanced postoperative care (navigator arm) or usual care. The randomization schedule was stored in a password protected file, accessible only to the study data manager (JEC). Patients were randomized by the data manager (JEC) the day after surgery in the order in which surgery was completed. We used randomly varying permutated blocks with varying block sizes to randomize patients within 8 strata. The strata included age (<65 vs. 65+), sex, and surgeon (high volume vs. low volume). Please refer to Figure 1 for a visual representation of the two study arms.

### Interventions

#### Enhanced postoperative management (navigator arm)

Patients randomized to the navigator arm received ten calls over the six months following their surgery: weekly for the first postoperative month (weeks 1, 2, 3, 4), biweekly for months two and three (weeks 6, 8, 10), and monthly for months four through six (weeks 14, 18, 22). In accordance with the principles of MI, these calls had no set script or agenda; the navigators did not give instructions and only provided advice when asked. On each call, the objective of the navigator was to elicit information from the patient including: what goals the patient would like to achieve (short- and/or long-term, depending on the call), why the patient would like to achieve those goals (specifically, the benefits of the goal), how the patient planned to achieve the goals (specific strategies), any barriers or possible challenges the patient anticipated (including how the patient might deal with such challenges), and why the patient felt they would be able to achieve these goals. Navigators also encouraged and congratulated the patient’s achievements, gave the patient the opportunity to air frustrations and concerns, and used reflection and open-ended questions to affirm the patient’s experiences and progress. When appropriate, the navigators provided the patient with information and encouraged the patient to contact his or her surgeon, physical therapist, or other appropriate healthcare provider for medical concerns. Table 2 provides a list of major techniques used by the navigators.

**Table 2:** **Navigation techniques**: example conversation excerpts^*^

	
**Developing goals:**	
*Eliciting ambivalence*	“Getting to the gym has been very challenging now that you've returned to work! You mentioned that you would like to get to the gym more often – why is that?”
**Discussing established goals**	
*Confidence ruler*	“On a scale of 1 to 10, how confident are you that you can walk without a cane?”
	“Why did you rate yourself at [X] rather than a [lower number]?”
*Asking about potential barriers*	“What challenges do you anticipate as you start thinking about returning to golf?”
**Patient is discouraged and/or did not achieve goal**	
*Affirmation and encouragement*	“I'm sorry to hear that you had to go to the hospital this week for pneumonia - I'm so glad you're feeling better! Despite the interruption to your recovery, it's wonderful that you achieved 108 degrees of flexion. Last time we spoke you were at 100 degrees – this is a huge improvement!”
**In response to clinical concerns**^**+**^	
*Referral to appropriate clinician*	“It sounds like you're dealing with a lot of swelling in the knee and want a sense of whether that is normal. Did you mention this to the physical therapist today? What did he/she have to say?”
	“You're having new sharp pains in the knee. Have you considered speaking your surgeon and his/her team? If you would like, I can provide you with the number.”
**Throughout conversation**	
*Asking permission*	“Do you mind if I ask why you originally decided to go through with the surgery?”
*Open-ended questions*	“Where would you like to see yourself one week from now?”; “How have your exercises been going the last few weeks?”
*Simple and complex reflection*	“It sounds like the flexion exercises are the most difficult for you and the pain is tremendous. At the same time, I'm hearing that you feel these are the most important for your recovery. Can you tell me more about the benefits of these exercises?”
*Summarization*	“I'm so happy to hear that since we last spoke your flexion has improved, and you've felt comfortable cutting back on the pain medications. It sounds like you feel ready for the transition to outpatient therapy. Swelling is still making improvement difficult for you, but icing and elevation have been helping, and you hope to continue that through the next week. I look forward to speaking with you next week and hearing how things go as you continue to practice your gait and try walking outside for the first time! Do you have any questions or anything else you'd like to discuss today?”

^*^All techniques, except “referral to appropriate clinician” were informed by the principles of motivational interviewing.

^+^Navigators were trained to recognize symptoms of serious adverse events (such as infection or pulmonary embolus), and were to instruct the patient to dial 911 in such cases.

Early navigator calls often focused on patient goals in relation to physical therapy (including adherence to the prescribed regimen, achievement of range-of-motion milestones, pain management through icing and medications), as well as short-term goals to return to the patient’s desired level of function (including household activities, improved mobility, and return to activities out of the home). As the patient moved along in recovery, focus often shifted toward patient’s longer-term goals for recovery (including athletic activities, participation in specific upcoming events, and commitment to regular physical activity). Table 3 lists common goals at each stage of the recovery process.

**Table 3:** **Common patient goals after total knee arthroplasty**^+^

	
**Weekly 1–4 (*postoperatively*)**	▪ Reduce pain, swelling; improve sleeping
	▪ Adhere to prescribed physical therapy routine
	▪ Improve range-of-motion (extension and flexion)
	▪ Move from walker → cane → no supportive device
	▪ Improve mobility; increase walking distance (getting out of the house, walking up and down stairs)
	▪ Reduce pain medications
**Weekly 6–10 (*postoperatively*)**	▪ Limit pain medications to evenings and before physical therapy sessions
	▪ Improve speed and stability of gait
	▪ Start driving again
	▪ Return to work
	▪ Walk up and down stairs “reciprocally” (“foot-over-foot”)
	▪ Improve range of motion (extension and flexion)
	▪ Fit physical therapy into increasingly busy routine
	▪ Increase time and comfort on stationary bicycle
	▪ Prepare for athletic goals (tennis, biking, walking, hiking, golf, etc.)
	▪ Return to regular daily walking, stretching, exercise routines
	▪ Adhere to prescribed physical therapy routine
**Weekly 14–22 (*postoperatively*)**	▪ Develop a regular routine that includes physical activity
	▪ Continue to strengthen leg muscles
	▪ Return to athletic activities (tennis, biking, walking, hiking, golf, etc.)
	▪ Manage co-morbid musculoskeletal conditions

^+^Each patient is unique and the trajectory of recovery can vary greatly; however, this table is intended to give a general sense of what many patients’ goals may be at different stages of recovery.

#### Navigator training

Prior to working with patients, care navigators studied the work on MI by its creators, Miller and Rollnick [14], and received personal training and coaching in MI techniques by an expert in MI with doctoral training in behavioral science and clinical epidemiology. Navigators observed inpatient and outpatient care (including physical therapy and outpatient follow-up with the surgeon) specific to post-TKA patients.

#### Usual care arm

Patients randomized to the usual care arm completed the standard of care rehabilitation protocol offered by BWH for TKA recipients. Usual care consists of immediate active or active-assisted range of motion and progressive gait training during the hospital stay. The majority of patients are discharged home with a prescribed physical therapy regimen and later transition to outpatient physical therapy. Often patients receive some form of therapy three to six months after surgery, with the exact number of visits varying among patients.

### Outcome measures

The primary outcome of the study was the difference between the two arms in functional improvement from preoperative (baseline) status to six months postoperatively, as measured by the difference in Western Ontario and McMaster Universities Osteoarthritis Index (WOMAC) function score. Secondary outcomes included quality-of-life improvements (as measured by EuroQol) and health care services utilization over the course of three and six months postoperatively.

### Assessments

#### Timing

All study participants received their initial assessment at the time of preoperative visit, usually occurring within two weeks prior to the date of surgery. Study participants were also assessed at three months after surgery and at the time of primary outcome assessment, six months postoperatively. These assessments were done by self-reported questionnaires.

#### Blinding

The principle investigator and the team responsible for patient enrollment and assessment were blinded to the patients’ study group assignments. Un-blinding personnel only took place in emergency situations. If un-blinding needed to occur, the study coordinator noted the un-blinded patient’s identification number, reason for un-blinding, person responsible for un-blinding study staff, and the list of persons now un-blind to the patient’s treatment.

### Noncompliance and termination

A patient’s participation was considered complete if the patient completed all study assessments through the six month time point. Patients were terminated if they cancelled their TKA, underwent a contralateral TKA within six months of their index procedure, or if their surgeon requested their early termination. In the case of contralateral TKA, patients assigned to the navigator arm continued to be followed by the navigator until their contralateral TKA was performed.

If a patient requested withdrawal from the navigator arm they were invited to submit their three and six month questionnaires, enabling the research team to track outcomes of those who declined participation in the intervention arm over the course of the study. Patients who were unwilling to continue with the study protocol altogether were considered early withdrawals. Patients who requested withdrawal from the study were asked standardized questions regarding their reason(s) for withdrawal including cancellation of surgery, too sick, too busy, no longer interested, or other.

There were no additional evaluations for withdrawn patients. At the time of early withdrawal, premature termination, or study completion, the research assistant mailed the patient a study completion letter which documented their withdrawal and confirmed that this would not affect the medical care the patient received at BWH.

### Adverse events

Adverse events included medical or surgical complications occurring within six months after index TKA. The clinician investigators on the AViKA team ascertained the seriousness of each adverse event and whether it was related to the trial. Adverse events were reported to the Partners IRB per IRB policies.

### Sample size and power

The principal outcome measure was the difference between arms in functional improvement as measured by the difference in the WOMAC function scale from baseline to six months. The sample size considerations were based on the ability of the study to detect a ten point difference between the care navigator and usual care arms. This difference is approximately one half of the standard deviation of change noted in observational pilot data. It is also in the range of the minimal clinically important difference in the WOMAC function scale among OA patients estimated by Angst et al. [15,16]. We adopted a Type I error rate of 5% and power of 80%. The sample size calculation took into account losses to follow-up, which we estimate at approximately 10% based on pilot data. We powered the study for one pre-planned subgroup analysis in which obese patients will be compared to non-obese patients via formal testing of the statistical significance of a multiplicative interaction between the intervention effect on obese and non-obese TKA recipients. On the basis of these considerations, we set the target sample size at 300 patients. With an anticipated 10% attrition rate we plan to enroll 345 subjects.

### Analytic approach

The primary analysis will be a comparison of six month changes in WOMAC function score between the care navigator and the usual care groups, using the “intention to treat” approach, in which subjects are analyzed in the group to which they were randomly assigned. We will also perform a secondary “intention to treat” analysis in which the dependent variable is a failure indicator defined as failing to achieve a minimal clinically important improvement in WOMAC function score (one half of the standard deviation) [15,16]. This analysis provides an estimate of efficacy at the level of the subject rather than the group. We will perform additional exploratory analyses to identify factors associated with outcome, including preoperative functional status and pain, catastrophizing, self-efficacy, and depression, as per prior literature [3-7]. If the intervention is associated with improvements in functional outcome, we will use general linear models to determine whether intervention effects are mediated by improvements in self-efficacy or catastrophizing.

In addition, a formal economic analysis is planned to determine the cost-effectiveness of the care navigator intervention as compared with usual care. The analysis will incorporate accepted principles of cost-effectiveness analyses including a societal perspective and discounting of all costs and health benefits [17].

### Data management

We managed study data in REDCap (Research Electronic Data Capture), a secure, web-based application designed to support data capture for research studies [18]. Data were entered into the REDCap database and exported to SAS version 9.2 for statistical analysis (SAS Institute, Cary NC). The hard copy questionnaires and medical chart abstractions are kept on site in a locked file cabinet.

The REDCap calendar function alerted study staff to follow-up questionnaires due to be sent. The follow-up window for the three and six month questionnaires were ± two weeks and ± four weeks, respectively. Mailings to patients included a cover letter, the follow-up questionnaire, and a pre-paid envelope with the BWH address preprinted for easy return. If a patient's questionnaire was not received within two weeks of the mailing, study staff made up to four phone calls to the patient to inquire on the status of the questionnaire. Data from returned questionnaires were immediately entered into the REDCap database and a remuneration check of $25.00 was issued to the study participant. If no contact was made with the patient and the questionnaire was not received by the end of the event window, the questionnaire was listed as not returned.

## Discussion

The AVIKA Navigator study was designed to address a critical question related to successful delivery of TKA: whether support from persons trained in motivational interviewing techniques leads to a more efficient and satisfactory rehabilitation process post-TKA, as measured by improvements in functional status between baseline and six months, and whether such support is particularly beneficial in persons with concomitant comorbidities, including obesity. The study is set to enroll 345 subjects in a tertiary clinical center between August, 2010 and December, 2013. This will be the largest clinical trial to date to examine the efficacy of a motivational-interviewing-based strategy to improve recovery following TKA. The cost-effectiveness analysis planned alongside the trial will address questions regarding the value of the intervention, as well as its budgetary impact for hospitals and payers. Given drastic increases in the utilization of TKA, efforts to improve recovery while controlling resource utilization are critical to ensure the clinical and economic value of TKA.

## Abbreviations

TKA: Total knee arthroplasty; OA: Osteoarthritis; MI: Motivational interviewing; AViKA: Adding Value in Knee Arthroplasty; RCT: Randomized controlled trial; BWH: Brigham and Women’s Hospital; LMR: Longitudinal medical record; ESP: Electronic surgical plan; IRB: Institutional review board; WOMAC: Western Ontario and McMaster Universities Osteoarthritis Index; REDCap: Research Electronic Data Capture.

## Competing interests

Dr. Wright is a paid consultant for Depuy Orthopedics, but does not have any competing interest with respect to this study. No other authors have potential competing interests to disclose.

## Authors’ contributions

EL, RG, JNK, and JW conceived and designed the experiments. EL, JEC, MED, LADF, JJZP, DJS, VL, BNR, DS, and JNK acquired the data. EL and JEC analyzed and interpreted the data. EL, JEC, MED, LADF, JJZP, BNR, and JNK wrote the manuscript. All authors revised the article critically for important intellectual content and approved the final version of the manuscript.

## Pre-publication history

The pre-publication history for this paper can be accessed here:

http://www.biomedcentral.com/1471-2474/14/290/prepub
